# *Ganoderma lucidum* polysaccharide extract inhibits hepatocellular carcinoma growth by downregulating regulatory T cells accumulation and function by inducing microRNA-125b

**DOI:** 10.1186/s12967-015-0465-5

**Published:** 2015-03-26

**Authors:** Aimei Li, Xuanyu Shuai, Zhijun Jia, Hangyu Li, Xiubin Liang, Dongming Su, Wanhua Guo

**Affiliations:** Department of Pathology, Nanjing Medical University, Nanjing, China; Department of Nuclear Medicine, The Affiliated Drum Tower Hospital of NanJing University, Zhongshan Road, Nanjing, 210008 China; Department of General Surgery, Shengjing Hospital Affiliated to China Medical University, Shenyang, China; Center of Cellular Therapy, The Second Affiliated Hospital of Nanjing Medical University, Nanjing, China; Center of Metabolic Disease Research, Nanjing Medical University, 140 Hanzhong Road, Nanjing, 210029 China

**Keywords:** *Ganoderma lucidum* polysaccharides, Hepatocellular carcinoma, Regulatory T cell, Effector T cell, miR-125b

## Abstract

**Background:**

*Ganoderma lucidum* polysaccharides (GLPS) have been used as traditional Chinese medicine for their properties of cancer prevention and immunomodulation. However, it is unclear whether GLPS has therapeutic effect on anti-hepatocellular carcinoma (HCC) *in vivo*. In this study, the effect of GLPS and their impact on the balance of regulatory T cell (Treg) and effector T cell (Teff) was measured in a model of hepatoma-bearing mice.

**Methods:**

The effect of GLPS and their impact on the balance of regulatory T cell (Treg) and effector T cell (Teff) were measured in a model of hepatoma-bearing mice. Real-time PCR detected the levels of MicroRNAs (miRNAs) and mRNA. The effects of Tregs on Teff proliferation were determined via suppression assay. The mircroRNA-125b (miR-125b) inhibitor was used to down-regulate miR-125b expression.

**Results:**

GLPS significantly suppressed tumor growth in hepatoma-bearing mice associated with an increase of the ratio of Teffs to Tregs. Moreover, GLPS eliminate Treg suppression of Teff proliferation with an increase in IL-2 secretion. Addition of GLPS to treat T cells inhibited Notch1 and FoxP3 expression through increase of miR-125b expression. In hepatoma-bearing mice, miR-125b inhibitor obviously abolished the effect of GLPS on tumor growth.

**Conclusions:**

This finding provides the novel evidence for GLPS on inhibition of HCC through miR-125b inhibiting Tregs accumulation and function.

## Introduction

Hepatocellular carcinoma (HCC) is one of the most lethal cancers worldwide, especially in China [1]. Due to chronic hepatitis infection and inflammation, the efficacy of chemoembolization or systemic therapies on HCC remains limited. Many researchers have found that CD4 + CD25+ regulatory T cells (Tregs) population increased in both the peripheral blood and tumor microenvironment in HCC patients, which correlates with a poor prognosis [2,3]. Tregs play a key role in impairing the anti-tumor immune response and helping tumor cells to escape from immune surveillance [4]. Therefore, targeting the number and function of Tregs may be a useful and effective tool for HCC therapies.

MicroRNAs (miRNAs) are small non-coding RNAs involved in modulating gene expression at the post-transcriptional level [5]. miRNAs play critical roles in several different physiological processes, such as cell proliferation, apoptosis, development, metabolism and differentiation [6]. Increasing amounts of evidence have demonstrated that alteration of miRNAs expression is directly implicated in the process of tumorigenesis. Some miRNAs acted as tumor suppressors and others exerted an oncogenic activity [7,8]. Among these miRNAs, miR-125b aberrant expression is involved in tumorigenesis and progression of numerous human cancers [9]. In HCC, miR-125b is significantly downregulated and exerts tumor-suppressive function [10]. Jia et al. showed that miR-125b expression was obviously decreased in HCC tissues and cells, which is the prediction of aggressiveness and poor prognosis of HCC [11].

*Ganoderma lucidum*, a traditional remedy, has been widely used as adjuvant of anti-tumor therapy in clinic [12]. Polysaccharides, one of the major categories of the bioactive ingredients of ganoderma lucidum, have the multiple biological activities such as improvement of host immune function, prevention of oxidative damage, protection of liver with little toxicity [13,14]. Recently, the inhibitory effect of *Ganoderma lucidum* polysaccharides (GLPS) on tumor has received great attention [15,16]. GLPS could inhibit the tumor growth and improve the immune function *in vitro* and *in vivo* [17]. The immune-modulating activities of GLPS were due to different mechanisms, such as enhancing the cytotoxic activity of NK cells, activating Dendritic cells (DCs), and promoting T helper 1 immune responses [18]. However, the molecular mechanism of GLPS on Tregs in HCC is not clear. Therefore, in the present study, we have investigated the effect of GLPS on anti-hepatocellular carcinoma *in vitro* and *in vivo*, and explored the underlying mechanisms associated with this activity.

## Method and material

### Ethics statement

Animals were treated humanely, using approved procedures in accordance with the guidelines of the Institutional Animal Care and Use Committee at Nanjing University. The study was approved by the Experimental Animal Ethics Committee at Nanjing University.

### Chemicals and reagents

RPMI 1640 and DMEM were obtained from GIBCO (Invitrogen Company). Fetal bovine serum (FBS) was from Hyclone (Logan, UT, USA). GLPS was purchased from Johnsun Mushroom (Hangzhou, China). PHA and MTT were purchased from Sigma Aldrich (St. Louis, MO, USA). Lipofectamine 2000 transfection reagent was obtained from Invitrogen Life Technologies (Grand Island, NY, USA). Anti-mouse CD4-fluorescein isothiocyanate (FITC) and anti-mouse CD25-phycoeritrin (PE) were obtained from eBioscience (San Diego, CA, USA). Rabbit-anti-β-catenin and rabbit anti-Notch1 were purchased from Santa Cruz Biotechnology (Santa Cruz, CA, USA). The Detergent Compatible (DC) Protein Assay kit was purchased from Bio-Rad Laboratories (Hercules, CA, USA). The miRNeasy Mini kit, the miScript Reverse Transcription kit and the miScript SYBR Green PCR kit were purchased from Qiagen (Hilden, Germany).

### Cell culture

The mouse hepatoma H22 cell line (syngenic to the Kunming strain of mice) and normal hepatic cell line L-02 were obtained from Chinese Academy of Sciences (Shanghai, China). H22 cells were cultured in complete RPMI 1640, and then 0.5 × 10^7^ cells (200 μL) were injected into the abdominal cavity of mouse. Seven days later, the intraperitoneal tumor cells were collected from the mouse with ascites tumor. L-02 cells were cultured in DMEM, supplemented with 10% FBS maintained at 37°C in a humidified incubator containing 5% CO_2_. Cultured cells were treated with GLPS (dissolved in PBS) in complete medium.

### Hepatoma-bearing mice

Kunming and BALB/c male mice were purchased from Shanghai Laboratory Animal Centre (Chinese Academy of Sciences, Shanghai, China). The HCC model of mice was performed according to a previous report [19]. Briefly, 200 μL (0.5 × 10^7^ cells) of H22 cells suspension was injected into the abdominal cavity of Kunming mouse. Ten days later, mice were randomly and equally divided into several groups (ten mice per group). To investigate the anti-tumor effect of GLPS, mice were divided into 5 groups: injection of PBS, 10 mg/kg GLPS, 50 mg/kg GLPS, 100 mg/kg GLPS and 200 mg/kg GLPS. To observe the role of miR-125b in anti-tumor of GLPS, mice were also divided into 4 groups: injection of negative control (5′-CAGUACUUUUGUGUAGUACAA-3′), miR-125b inhibitor (5′-UCACAAGUUAGGGUCUCAGGGA-3′), GLPS + negative control, and GLPS + miR-125b inhibitor. Cholesterol-conjugated miR-125b inhibitor and negative control oligonucleotide (NC) were purchased from RiboBio (Guangzhou, China). GLPS in 0.5 ml of PBS was administered by intraperitoneal injection (i.p.) every two days for 4 weeks. In GLPS + miR-125b inhibitor group, miR-125b inhibitor was pretreated for 24 hours and then injected with GLPS. NC and miR-214 inhibitor (10 nmol) in 0.1 ml of saline buffer was administered intratumorally every 2 days for 4 weeks. Tumor growth was assessed two times a week and volume (V) was calculated using the formula V = 1/2 × length × (width)^2^.

### Isolation of tumor-infiltrating lymphocytes (TILs)

TILs were isolated from the tumors according to previous report [20]. Briefly, small pieces of solid tumors tissue was digested with an enzyme cocktail containing 2% fetal bovine serum, 0.5 mg/ml collagenase A (Roche), 0.2 mg/ml hyaluronidase, type V (Sigma) and 0.02 mg/ml DNase I (Sigma) per 0.25 g of tumor tissue. The cell suspensions were filtered through a cell strainer (70 μm, Becton Dickinson, CA, USA), and then, they were washed with 2% FBS in RPMI 1640. After lysed red blood cells, the cell clumps were removed by 40%/70% Percoll gradient centrifugation and centrifuged at 400 g. The nonadherent lymphocytes were harvested and CD4+ lymphocytes were isolated by Dynabeads M-450 CD4 according to the manufacturer’s instruction. These CD4+ lymphocytes were further isolated into CD25+ and CD25- lymphocytes by Dynabeads (Dynal Biotech ASA, Oslo, Norway). CD4 + CD25+ T cells and/or CD4 + CD25- T cells were added different concentrations of GLPS, and cultured for 72 h, at 37°C and 5% CO2.

### Isolation of lymphocytes from peripheral blood

EDTA-K2 anticoagulated blood samples were collected from hepatoma-bearing mice. Monocytes were isolated from the peripheral blood by Ficoll-Paque (Pharmacia, Piscataway, NJ) density centrifugation. The monocytes were resuspended in RPMI-1640 medium and plated on a 6-well plate. After 2 hours of incubation at 37°C, the nonadherent lymphocytes were harvested and subsequently cultured.

### Isolation of mouse naïve T cells and *in vitro* T cell differentiation

Splenic CD4+ naïve T cells from wild type Kunming mice were isolated using the CD4+ T cell Isolation Kit (Miltenyi Biotec, Bergisch Gladbach, Germany) following the manufacturer’s instructions. Isolated naïve T cells were added different concentrations of GLPS, and cultured in RPMI-1640 with 10% FCS with 2 ng/ml of mouse TGF-β1, 1500 units/ml of mouse IL-2, and 100 nM of retinoic acid (iTreg polarizing condition) for 7–10 days.

### Flow cytometric analysis

TILs were harvested from the tumors and the lymphocytes were isolated from peripheral blood. For intracellular staining, monoclonal antibodies to surface molecules, including Anti-CD4-FITC and anti-CD25-PE were used to stain surface markers. The percentage of CD4 + CD25- and CD4 + CD25+ T cells was computed using Cell-Quest software (Becton Dickinson).

### Suppression assay

Suppression assays were performed by seeding an equal number of CD4 + CD25− T cells and CD4 + CD25+ T cells (1 × 10^5^/well) into 96-well plates in the presence of PHA (5 μg/ml) and cultured with or without GLPS for 72 h. After 72 h, [3H]-thymidine (Amersham Biosciences) was added (1 μCi/well) for 18 h. Next, [3H]-thymidine incorporation was measured on a β-scintillation counter. Results were expressed as mean cpm ± SE.

### Lymphocyte proliferation assay

The [3H]-thymidine incorporation assay was performed according to previous report [21]. Briefly, CD4 + CD25− TILs (1 × 10^5^ /well) were seeded into 96-well plate in the presence of PHA and cultured with or without GLPS. After 72 h, each well is pulsed with 1 μCi [3H]-thymidine for 18 h. Cells were harvested, and [3H]-incorporation measured using a β-scintillation counter. Results were expressed as the mean cell proliferation in counts per minute (cpm) ± standard error (SE).

### Oligonucleotides and Cell Transfection

Jurkat T cells were seeded in 6-well or 24-well plates and transfected using Lipofectamine 2000 (Invitrogen, Carlsbad, CA, USA) according to the manufacturer’s instructions. For the knockdown of miR-125b, anti-miR-125b or a negative-control anti-miRNA (anti-NC) was used at the concentration of 100 nM. The cells were harvested 24 h after the transfection.

### Quantitative real-time PCR (Q-PCR) analysis

Mature miRNAs were isolated and purified using Trizol reagent (Invitrogen, USA), according to manufacturer’s protocol. The levels of miRNAs (miR-126, miR-155, miR-146a, miR-224, miR-150 and miR-125b) were quantified by using a TaqMan PCR kit (Applied Biosystems, Foster City, CA, USA). Commercially available Taqman primers and probes, including 2 unlabeled PCR primers and 1 FAMTM dye-labeled TaqMan MGB probe were used for all the targets. Real-time PCR was performed using LighteCycler480 II Sequence Detection System (Roche, Basel, Switzerland). All reactions, including no-template controls, were performed in triplicate. After the reaction, the CT values were determined using fixed threshold settings. In order to calculate the differences of expression level for each target among samples, the 2^-ΔΔCT^ method for relative quantitation was used. All data were analyzed using U6 small nuclear RNA as an internal normalized reference.

The mRNA expression of North1 and FoxP3 was performed using SYBR GREEN PCR Master Mix (Applied Biosystems). The specific primers were as follows: North1, 5′- CCGGTGAGACCTGCCTGAAT-3′ (forward) and 5′- GCACTTGTACTCCGTC AGCG -3′ (reverse); FoxP3, 5′-ACTGACCAAGGCTTCATCTGTG-3′ (forward) and 5′–GGAACTCTGGGAATGTGCTGT-3′(reverse); GAPDH, 5′-TGAAGCAGGCA TCTGAGGG-3′ (forward) and 5′-CGAAGGTGGAAGAGTGGGAG-3′ (reverse). All data were analyzed using GAPDH gene expression as an internal standard.

### Western blot analysis

Jurkat T cells were lysed with ice-cold lysis buffer containing: 50 mmol/l Tris–HCl, pH 7.4; 1% NP-40; 150 mmol/l NaCl; 1 mmol/l EDTA; 1 mmol/l phenylmethylsulfonyl fluoride; and complete proteinase inhibitor mixture (one tablet per 10 ml; Roche Molecular Biochemicals, Indianapolis, IN, USA). Protein concentration in the cell lysate was quantified using the DC protein assay kit. Following protein content determination, western blot analysis was performed.

### ELISA analysis

Analysis of IL-2 was performed using an ELISA kit according to manufacturer’s protocol. Briefly, regulatory T cells and effector T cells were incubated in a 96-well plate with different concentrations of GLPS. Production of IL-2 was normalized to protein concentrations using the DC protein assay kit (Bio-Rad).

### MTT assay

Cell viability was determined using MTT [3-(4,5-dimethylthiazol-2-yl)-2,5-diphenyltetrazolium bromide] assays. The cells were seeded in 96-well dishes at 1 × 10^4^ to 2 × 10^4^ cells per well and pretreated with or without GLPS for 24 h. Each well was supplemented with 10 μl MTT (Sigma Aldrich) and incubated for 4 h at 37°C. The medium was subsequently removed, and 150 μl DMSO (Sigma Aldrich) were added to solubilize the MTT formazan. The optical density was observed at 490 nm.

### Statistical analysis

Statistical analysis was performed with statistical analysis software SPSS 13.0 software. Statistical analyses were performed using either an analysis of variance (ANOVA) or Student’s *t*-test. Data were expressed as mean ± standard deviation. *P* < 0.05 was considered to be significant.

## Results

### GLPS inhibited hepatoma growth *in vivo*

**Figure 1:**
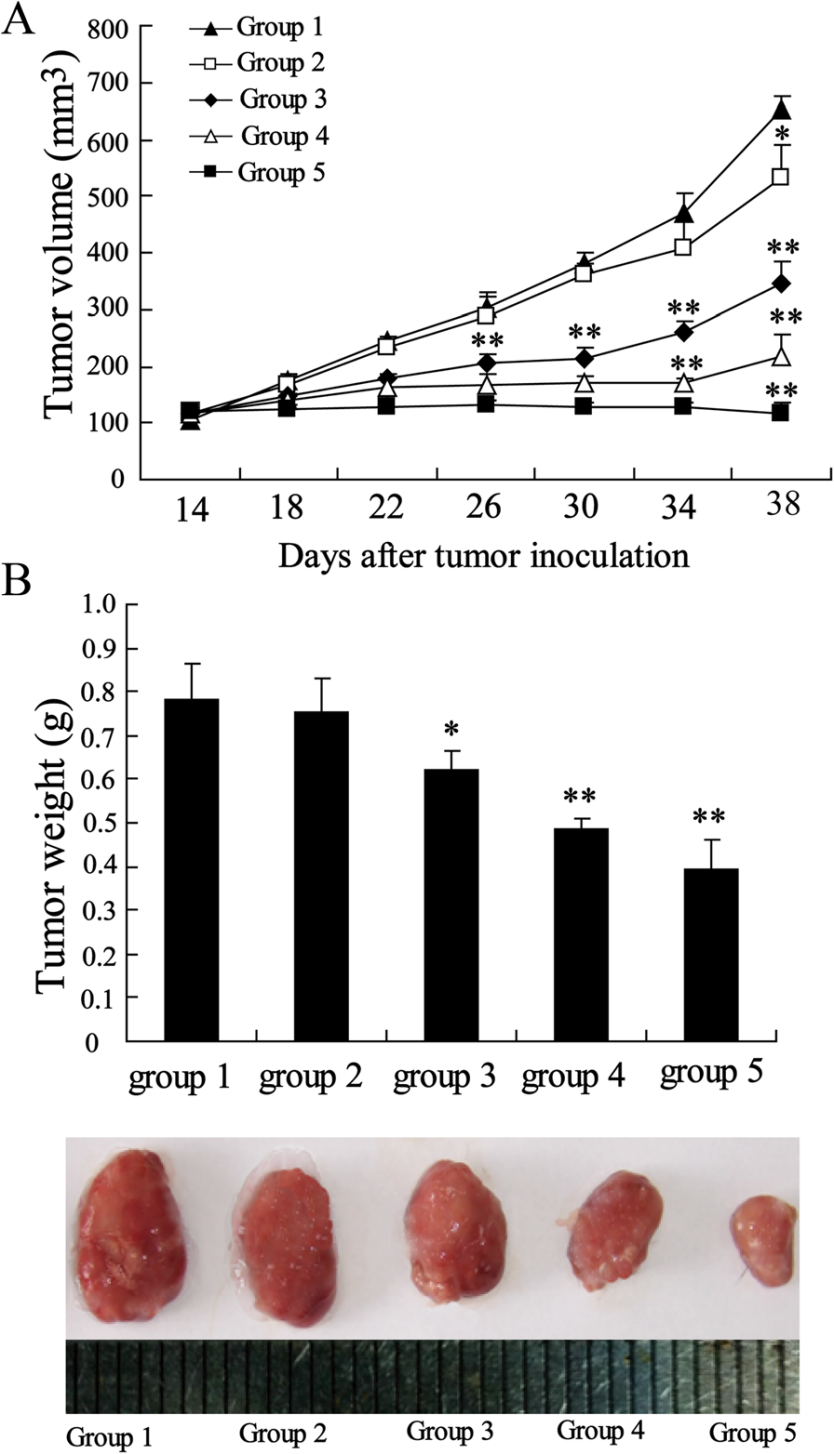
**GLPS inhibited hepatoma growth** ***in vivo*** **.** The hepatoma-bearing mice were treated with 0 mg/kg (group 1), 10 mg/kg (group 2), 50 mg/kg (group 3), 100 mg/kg (group 4) and 200 mg/kg (group 5) GLPS by i.p. injection. **(A)** Tumor sizes on each mouse were monitored 2 times per week. **(B)** Tumor weights were measured on day 38 following H22 cells cell inoculation and the images of tumors from different treatment group were shown. *P < 0.05 and **P < 0.01 indicate significant differences from group 1.

To evaluate the anti-HCC activity of GLPS *in vivo*, the hepatoma-bearing mice model was established. After administration of GLPS at 10, 50, 100 and 200 mg/kg in hepatoma-bearing mice by i.p. every two days for 4 weeks, the tumor volume was significantly reduced by 18.3%, 47.08%, 66.92% and 81.9%, respectively. As shown in Figure 1A, the mean tumor volume on day 38 following H22 cells cell inoculation, which was 652 ± 25.51 mm^3^ in control group, was reduced to 118 ± 17.35 mm^3^ in the GLPS treatment group (200 mg/kg). Besides, GLPS treatment significantly decreased the weight of tumors in a dose-dependent manner (Figure 1B).

### GLPS shifts the intratumoral regulatory T cell (Treg) to effector T cell (Teff) balance

**Figure 2:**
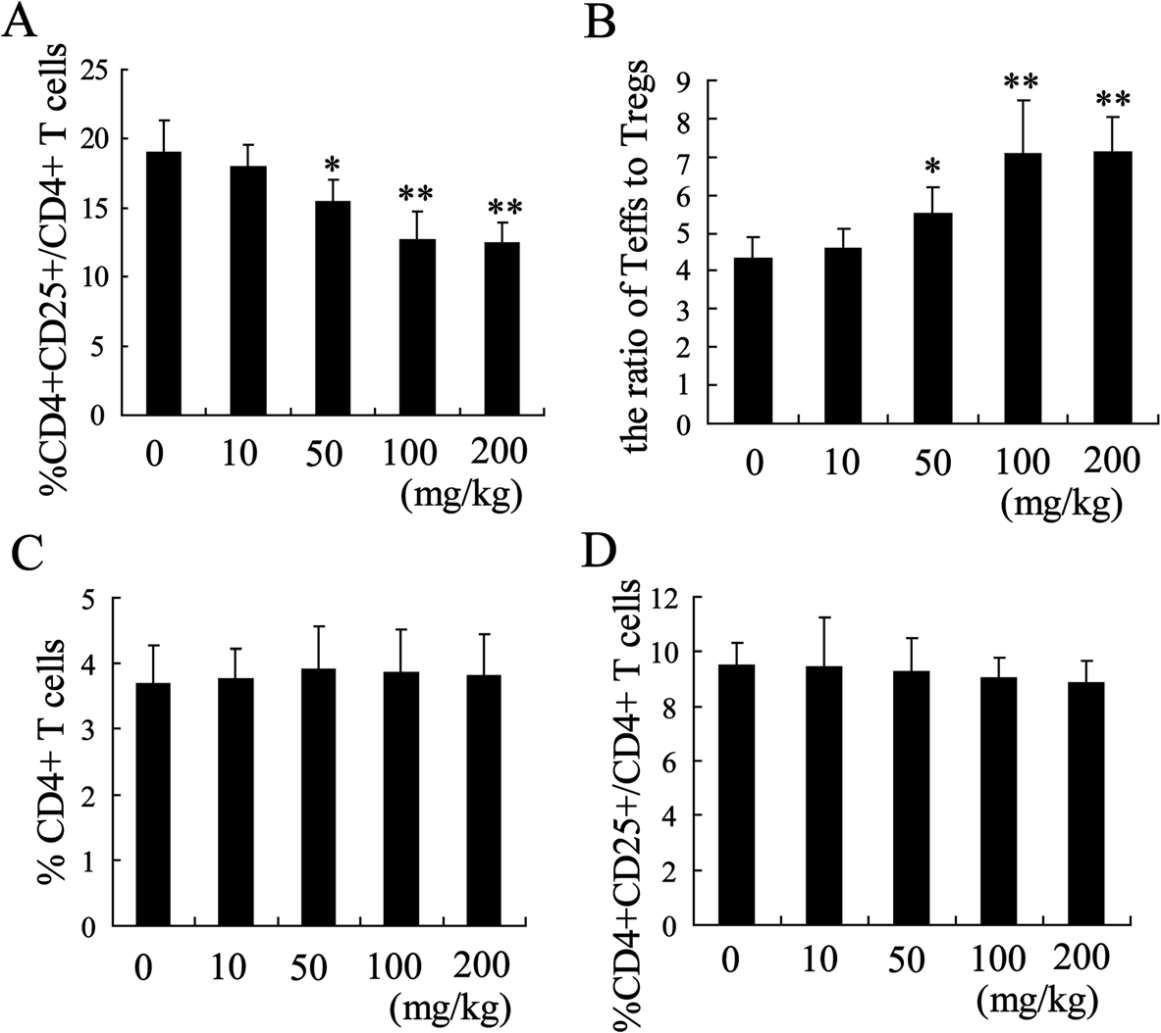
**GLPS shifts the intratumoral regulatory T cell (Treg) to effector T cell (Teff) balance. (A)** The ratio of intratumoral Tregs to CD4+ cells was measured by flow cytometric analysis from hepatoma-bearing mice treated with 0 mg/kg, 10 mg/kg, 50 mg/kg, 100 mg/kg and 200 mg/kg GLPS by i.p. injection. **(B)** The ratio of Teff to Treg was calculated. **(C)** The percentage of CD4+ T cells in tumor was detected by flow cytometric analysis. **(D)** The population of Tregs T cells in the peripheral blood of hepatoma-bearing mice was measured with flow cytometric analysis *P < 0.05 and **P < 0.01 indicate significant differences from hepatoma-bearing mice treated with 0 mg/kg GLPS.

To explore the mechanism of GLPS inhibiting tumor growth in the hepatoma-bearing mice, we investigated the impact of GLPS on the balance of Treg (distinguished as CD4 + CD25+) and Teff (distinguished as CD4 + CD25-) in the tumor. After injection of GLPS for 4 weeks, the ratio of intratumoral Tregs was dose-dependently decreased but that of Teffs was increased, which resulted in an increase of the ratio of Teffs to Tregs (Figure 2A and B). It should be noted that the percentage of total CD4+ T cells in tumors remained unchanged (Figure 2C). Besides, the frequency of Tregs in peripheral blood from GLPS-injected mice was slightly lower than that from PBS-injected mice (Figure 2D).

### GLPS decreases Treg-mediated suppression on Teff

**Figure 3:**
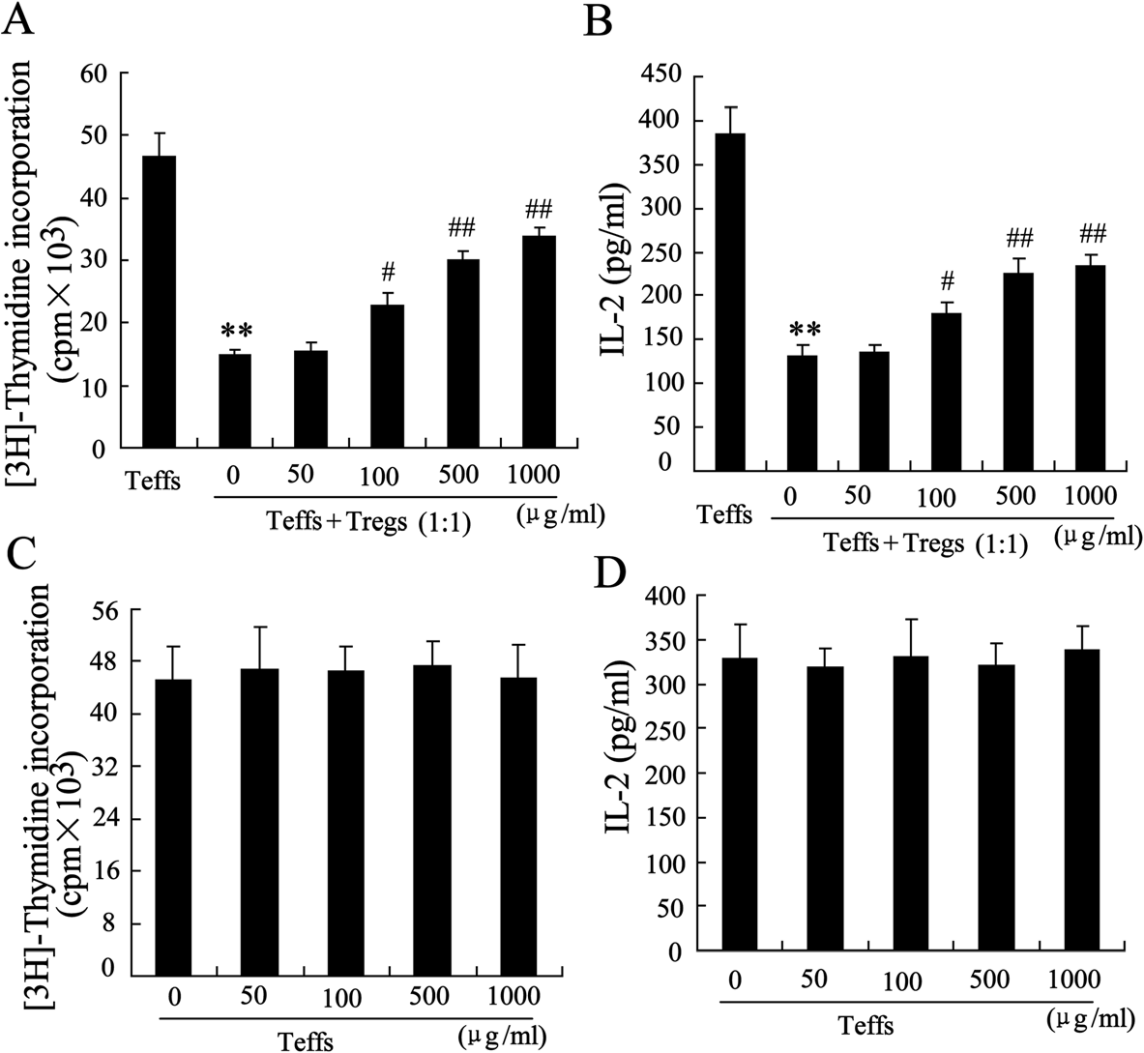
**GLPS decreases Treg-mediated suppression on Teff.** Tregs and Teffs isolated from the hepatoma-bearing mice. The addition of GLPS to the cocultures for 72 h and suppression assays were performed **(A)** and the secretion of IL-2 were determined **(B)**. **(C)** GLPS treated Teffs from the hepatoma-bearing mice for 72 h and proliferation assay was performed. **(D)** IL2 content in the supernatants from the proliferation assay was determined by ELISA. **P < 0.01, indicate significant differences from Teffs, # P < 0.05 and ## P < 0.01, indicate significant differences from 0 mg/kg (μg/ml) GLPS.

It has been confirmed that Tregs have an immunosuppressive effect on Teffs [22]. To determine whether GLPS treatment had an effect on Treg activity, we performed the suppression assay of Tregs *in vitro*. Briefly, Tregs and Teffs isolated from the hepatoma-bearing mice were co-cultured (1:1) in 96-well plate. The addition of GLPS to the cocultures for 72 h significantly decreases Tregs-mediated suppression on Teffs proliferation and increases IL-2 secretion in dose-dependent manner (Figure 3A and B). We also performed proliferation assays to evaluate the direct impact of GLPS on Teffs responses. To our surprise, GLPS at all tested concentrations did not stimulate Teffs proliferation without Tregs (Figure 3C). In addition, the supernatants from the proliferation assays showed GLPS did not affect the release of IL-2 from Teffs alone (Figure 3D).

### miR-125b was implicated in the effect of GLPS on intratumoral Tregs

**Figure 4:**
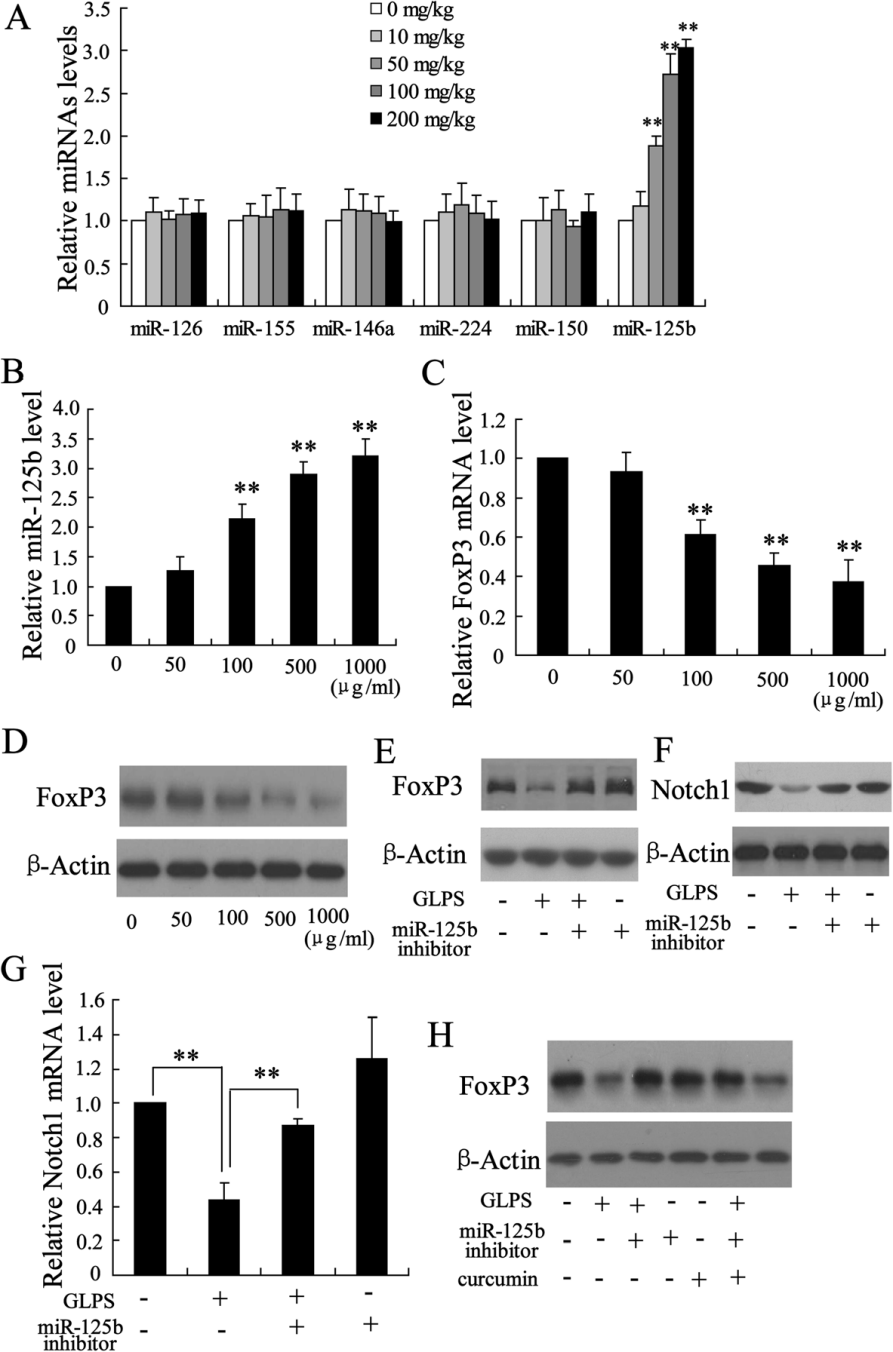
**miR-125b was implicated in the effect of GLPS on intratumoral Tregs. (A)** The expression of many miRNAs including miR-126, miR-155, miR-146a, miR-224, miR-150 and miR-125b was measured in Tregs from hepatoma-bearing mice injected with GLPS. **P < 0.01, indicate significant differences from Tregs isolated from hepatoma-bearing mice injected with 0 mg/kg GLPS. **(B)** The expression of miR-125b was determined in naïve T cells under iTreg polarizing condition with various concentrations of the GLPS for 96 h. **P < 0.01, indicate significant differences from 0 μg/ml GLPS treatment. The mRNA **(C)** and protein **(D)** levels of FoxP3 were measured in naïve T cells under iTreg polarizing condition with various concentrations of the GLPS for 96 h. **P < 0.01, indicate significant differences from 0 μg/ml GLPS treatment. After transfected with miR-125b inhibitor for 24 h, naïve T cells under iTreg polarizing condition were treated with GLPS (100 μg/ml) for 96 h. Then, the protein levels of FoxP3 **(E)** and Notch1 **(F)**, and the mRNA level of Notch1 **(G)** were determined. **P < 0.01, indicate significant differences from the respective control groups. **P < 0.01, indicate significant differences from the respective control groups. **(H)** After transfected with miR-125b inhibitor for 24 h, naïve T cells under iTreg polarizing condition were treated with GLPS (100 μg/ml) or GLPS (100 μg/ml) + curcumin (30 μmol/L). Then, the FoxP3 protein level was determined.

To further explore the mechanism of GLPS mediating the alteration of Treg to Teff balance in the HCC tissues, we measured the expression of many miRNAs including miR-126, miR-155, miR-146a, miR-224, miR-150 and miR-125b, which may be involved in Treg cell development [23]. Among these miRNAs, miR-125b was significantly increased in intratumoral Tregs from hepatoma-bearing mice injected with GLPS (Figure 4A).

To explore the possible role of miR-125b on induction and function of Tregs, we measured the miR-125b expression in naïve T cells under iTreg polarizing condition with various concentrations of the GLPS. After 96 h of treatment, the expression of miR-125b was significantly up-regulated in a dose-dependent manner (Figure 4B). When T cells were stimulated with GLPS, the levels of FoxP3 expression dose-dependently decreased (Figure 4C and D). The role of miR-125b in the effect of GLPS on Tregs was further investigated. Through depleting miR-125b in T cells using a miR-125b inhibitor, the decrease of FoxP3 expression induced by GLPS was abolished (Figure 4E), suggesting that the inhibition of Tregs by GLPS might act through increasing miR-125b expression.

It has been demonstrated that Notch1, a target of miR-125b, plays an important role in cell growth and apoptosis of HCC [24,25]. The effect of GLPS on Notch1 expression in T cells was measured. As shown in Figure 4F, GLPS treatment significantly down-regulated Notch1 expression, which was restored by the miR-125b inhibitor (Figure 4G). In addition, treatment with curcumin, a known Notch1 inhibitor, could reverse the effect of miR-125b inhibitor on FoxP3 expression in T cells exposed to GLPS (Figure 4H). These data indicate that GLPS could decrease FoxP3 levels by miR-125b down-regulation of Notch1 expression.

### miR-125b plays an important role in anti-tumor effect of GLPS

**Figure 5:**
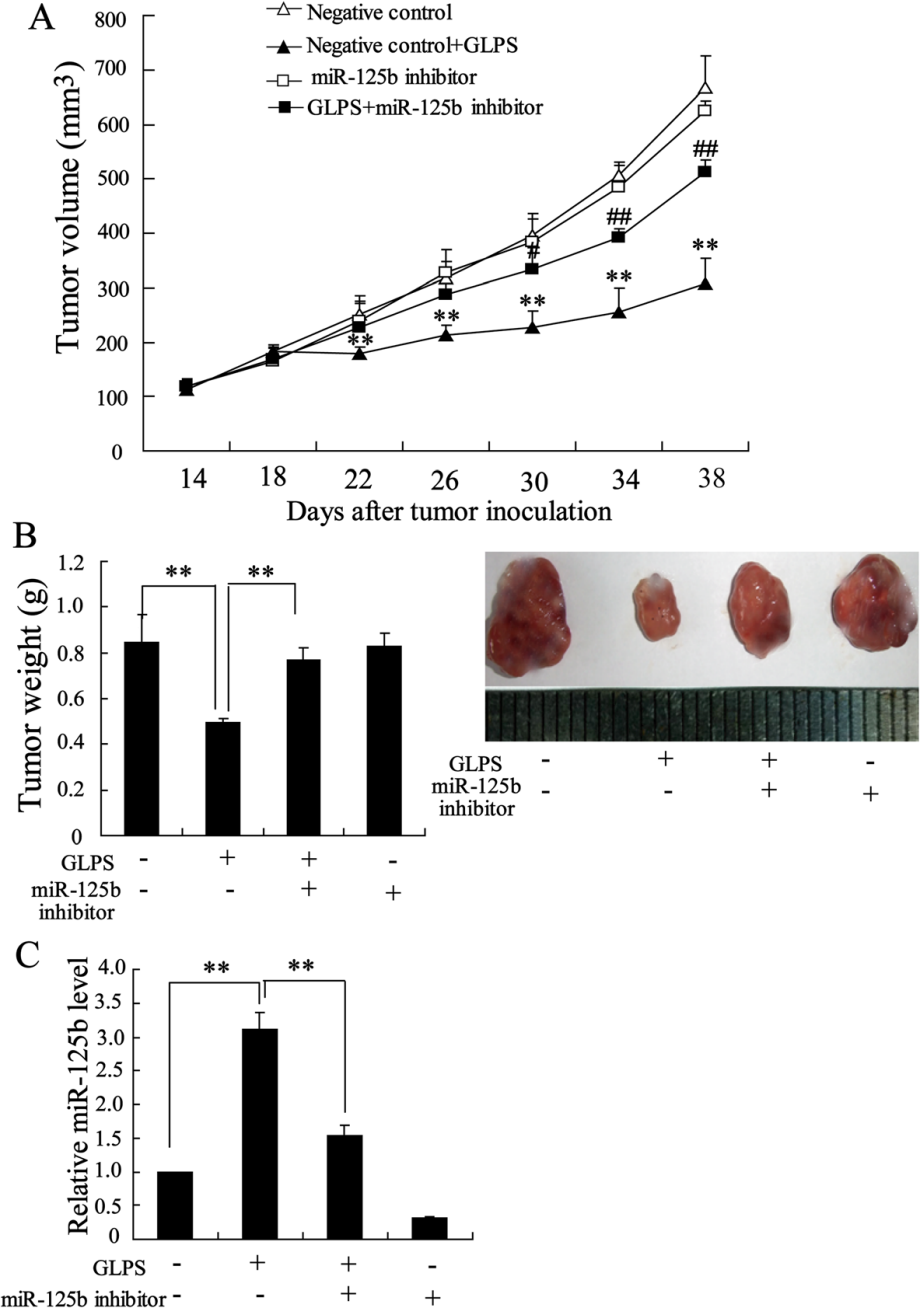
**miR-125b plays an important role in anti-tumor effect of GLPS.** For down-regulation of miR-125b, miR-125b inhibitor was injected intratumorally before GLPS administration. Twenty-four hours after the miR-125b inhibitor (10 nmol) injection, hepatoma-bearing mice were treated with 50 mg/kg GLPS or same volume of PBS by i.p. injection. **(A)** Tumor sizes on each mouse were monitored 2 times per week. **(B)** Tumor weights were measured and the images of tumors from different treatment group were shown. **(C)** The expression of miR-126b in Tregs from hepatoma-bearing mice were measured. **P < 0.01 indicated significant differences from negative control injection. # P < 0.05 and ## P < 0.01, indicate significant differences from negative control + GLPS.

To determine whether miR-125b was implicated in the anti-tumor effect of GLPS, the cholesterol-conjugated miR-125b inhibitor was administered into the hepatoma-bearing mice. After intratumoral injection of miR-125b inhibitor, the tumor volume and weight exhibited a slight increase. However, miR-125b inhibitor obviously restored the effect of GLPS on tumor growth (Figure 5A and B). In addition, the expression of miR-125b was obviously down-regulated in Tregs from hepatoma-bearing mice injected with GLPS and miR-125b inhibitor (Figure 5C). These results suggest that miR-125b plays an important role in the effect of GLPS on inhibition of HCC tumor growth.

### The effect of GLPS on H22 and L-02 cells viability

**Figure 6:**
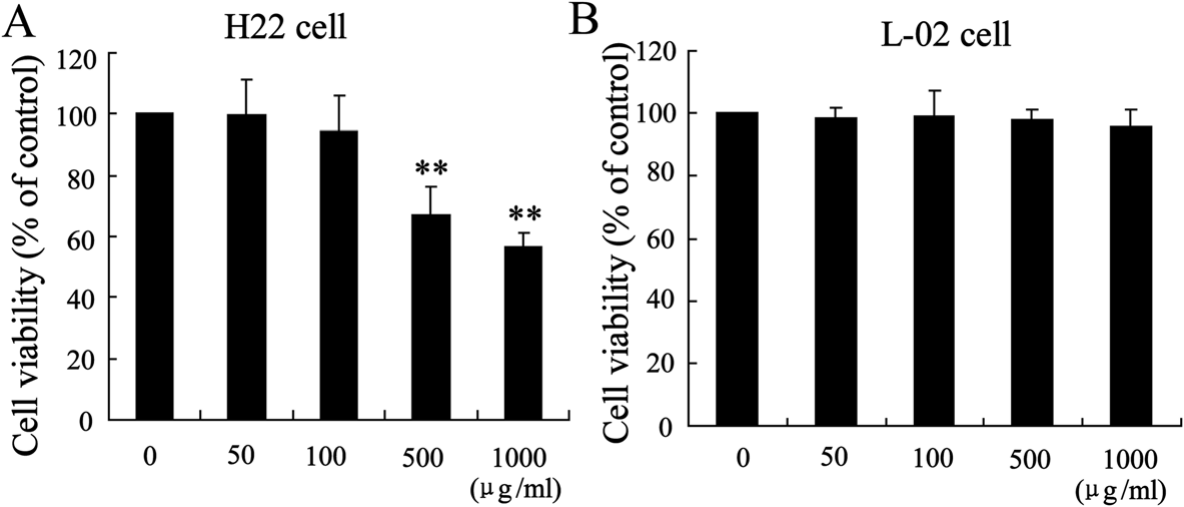
**The effect of GLPS on H22 and L-02 cells viability.** H22 cells **(A)** and L-02 cells **(B)** were incubated with the indicated concentration of GLPS for 48 h, and the cell viability was measured using a MTT assay. **p < 0.01, versus vehicle alone-treated cells.

It has been reported that GLPS induced apoptosis of HepG2 cells with high dosage [26]. To evaluate the potential effects of GLPS on the mouse hepatoma H22 cell and normal hepatic cell line L-02 viability, MTT assays were preformed. As expected (Figure 6), GLPS did not significantly suppress H22 cell viability at 50–100 μg/ml, while GLPS could inhibit cell viability at higher concentrations (>100 μg/ml). High dose of GLPS (1000 μg/ml) had no effect on L-02 cell viability, which indicate normal hepatic cells showed more resistance to the cytotoxicity effect of GLPS.

## Discussion

*Ganoderma lucidum* polysaccharides (GLPS), the traditional Chinese medicine, are effective in tumor therapy with low toxicity [27]. GLPS possess anti-tumor effects on many cancers through suppression of tumorigenesis, inhibition of tumor growth and metastasis, and modulation of immune cells [28]. In this study, the mechanism of GLPS on anti-HCC *in vivo* and *in vitro* was explored. First, we demonstrated that GLPS could significantly suppresse tumor growth in hepatoma-bearing mice associated with an increase of the ratio of Teffs to Tregs. GLPS eliminated CD4 + CD25+ Treg suppression of CD4 + CD25- Teff proliferation with an increase in IL2 secretion. Second, we showed that inactivation of tumor-infiltrating Tregs could abolish the antitumor activity of GLPS. Third, we also showed that miR-125b was implicated in the effect of GLPS on intratumoral Tregs and played an important role in anti-tumor effect of GLPS. It should be noted that GLPS had no effect on the weight of hepatoma-bearing mice (data not shown) and GLPS was little cytotoxic to normal hepatic cell line L-02 (Figure 6), which showed the safety of GLPS to treat HCC.

Regulatory T cells infiltrating the tumor play an important role in tumor immune evasion and become the main obstacle for successful HCC immunotherapy [29]. Increased the population of Tregs in both the peripheral blood and tumor has been shown to correlate with a poor prognosis in patients with HCC [30]. As a result, targeting the number and function of Tregs has been the target for HCC therapies. We provided a direct demonstration that GLPS treatment may definitely interfere with Treg accumulation and function *in vivo*. After injection of GLPS for 4 weeks, we established that the percentage of intratumoral Tregs was decreased in a dose-dependent manner (Figure 2A). The addition of GLPS to the cocultures of Tregs and Teffs resulted in a loss of Treg immunosuppressive properties (Figure 3A). However, GLPS at all tested concentrations did not stimulate Teffs proliferation without Tregs. These results demonstrated that GLPS suppressed liver tumor growth in a direct way via decreasing Tregs accumulation and activation.

Previous studies showed Notch1 signaling pathway played an important role in Treg differentiation and suppressor function through regulating FoxP3 expression [31]. Our data exhibited that GLPS treatment significantly down-regulated Notch1 expression in T cells, which indicated that Notch1 may be involved in GLPS immunomodulatory function. Further study showed that treatment with Notch1 inhibitor could reverse the effect of miR-125b inhibitor on FoxP3 expression in T cells. This suggested that Notch1 signaling was involved in GLPS-inhibited FoxP3 expression in T cells. It has been noted that TGFβ-smad pathway play a key role in the Treg and Teff balance [32]. We found that GLPS had no effect on the levels of TGF-β and p-Smad2/3 (data not shown), which indicated that GLPS downregulated regulatory T cells accumulation and function in a TGF-β-Independent Manner.

It has been demonstrated miR-125b expression was down-regulated in HCC, which is the prediction of aggressiveness and poor prognosis of HCC [33]. In this study, we found that miR-125b was implicated in the effect of GLPS on anti-HCC. First, miR-125b was significantly up-regulated in intratumoral Tregs from hepatoma-bearing mice injected with GLPS. Second, miR-125b expression displayed an increase in T cells treated with GLPS. Third, Notch1, a target of miR-125b, was down-regulated by GLPS. The present study, we have demonstrated that inhibition of miR-125b expression could attenuate GLPS-induced decrease of Notch1 level. Last, the effect of GLPS on suppression of tumor growth was reversed by miR-125b inhibitor. All these lines of evidence strongly suggested that GLPS exerted anti-HCC activity through up-regulation of miR-125b expression.

## Conclusion

GLPS effectively suppressed tumor growth in hepatoma-bearing mice. The decrease of Tregs accumulation and inactivation associated with GLPS administration was due to increase of miR-125b expression, which resulted in inhibition of Notch1 signaling pathway and FoxP3 expression. Overall, these findings have revealed the molecular mechanisms of GLPS treatment with HCC.
